# Supplementary material for: Improving cardiovascular health in patients with an abdominal aortic aneurysm: development of the cardiovascular risk reduction in patients with aneurysms (CRISP) behaviour change intervention
Source: Pilot Feasibility Stud. 2024 Jan 29;10:17. doi: 10.1186/s40814-024-01445-z (PMC10823620; doi:10.1186/s40814-024-01445-z)
Supplement: Supplementary file 1 — Additional file 1: Appendix 1. Scaffolding Questionaire. [file 40814_2024_1445_MOESM1_ESM.docx]

**SCAFFOLDING QUESTIONNAIRE**

**Intervention-development questions for health professionals: Cardiovascular Risk reduction In the NHS abdominal aortic aneurysm (AAA) screening programme: a co-developed cardiovascular prevention intervention (the CRISP study).**

This research is funded by the National Institute for Health Research (NIHR)

The CRISP study aims to develop an intervention which will help reduce cardiovascular risk in men with AAA. This intervention will be delivered alongside the existing AAA screening/surveillance programmes in the NHS. Following a few quick questions about you and your profession, this “scaffolding” questionnaire will seek your views about the priorities and content of our planned intervention.

**The questions relate to 3 KEY AIMS. Please add your responses in the space provided below.**

You can contact the research team on e-mail xxxxxxxxxxxxxxxxx at any time with your questions or feedback

***Some questions about you***

What is your healthcare profession/job title? *e.g. Sister Vascular Surgery or Public Health Doctor*

What is your role with respect to management of AAA?

Where are you based (which region)?

**Please now navigate through the three aims of this questionnaire (see next pages)**

**AIM 1: To develop our specific targets for the intervention i.e. what behaviour changes could reduce cardiovascular risk in people with AAA?**

Listed below are a number of potential **components of the intervention**, which are based on prior feedback from patients with AAA, their families and some experts in cardiovascular care. Please tell us what you think about each one. The delivery of the intervention will start when someone has been diagnosed with an AAA once they have attended AAA screening. Based on our prior research most men found to have an AAA or who are currently in AAA surveillance in the NHS do not receive adequate cardiovascular preventative care/medication. The separate components are highlighted in bold letters and underlined.

**Medication review** (to check all the patient’s medications are correctly prescribed and that any medications prescribed don’t affect each other or have side effects that could be prevented. Also to check if the patient has any problems organising or taking his medications). As per NICE guidance someone with an AAA should be offered an antiplatelet agent, a statin, and medication to reduce blood pressure (if relevant).

Should medication review be a component of the proposed intervention? Yes/No

If yes, who should undertake the medication review? *For example, GP, Pharmacist, Vascular Nurse*

To what extent does this already happen (please say who delivers this/in what circumstances) based on your experience in the NHS AAA screening and surveillance programmes?

How are prescriptions for medication given to individuals with AAA in your experience (i.e. does the GP give the patient a written prescription or does the AAA screening programme provide the patient with a prescription)?

Who checks the repeat prescriptions of individuals with AAA in your experience (GP, electronic service, pharmacist at the GP practice, the AAA surveillance programme)?

Please add any additional comments that you may have relating to medication(s).

***Prescription of antiplatelet agents and statins in specific (part of medication review component)***

Do you believe that the prescription of antiplatelet agents is currently sufficient in AAA screening/surveillance?

Do you believe that the prescription of statins is currently sufficient in AAA screening/surveillance?

Which antiplatelet agent (if any) is offered to individuals with AAA in your practice or region? (i.e. Clopidogrel or Aspirin or other agent)

Is there a preferred statin within your service?

Are lipid levels tested before prescribing statins in your service?

At what point in the care pathway does the prescription of statins takes place?

Is there any follow-up with the patient or the patient’s GP with regard to anti-platelets?

Is there any follow-up with the patient or the patient’s GP with regard to statins?

Please add any additional comments that you may have regarding antiplatelets or statins.

**Review of cardiovascular health and cardiovascular risk factors**

Should this be a component of the proposed intervention? Yes/No

If yes, who should deliver the CV risk review? *For example, GP, Pharmacist, Vascular Nurse*

To what extent does this already happen (please say who delivers this/in what circumstances) based on your experience in the NHS AAA screening and surveillance programmes?

Please add any additional comments that you may have relating to cardiovascular health/risk-factor review.

**Support for smoking cessation** (if applicable)

Should this be a component of the proposed intervention? Yes/No

If yes, who should try and promote smoking cessation? *For example, GP, Pharmacist, Vascular Nurse*

What local guidance or support is available (e.g. is a stop smoking clinic available where you are based)?

Please add any additional comments that you may have relating to smoking cessation.

**Advice on increasing physical activity** (if applicable)

Are there any safety considerations or counter-indications you are aware of for recommending low to moderate intensity exercise for people with AAA?

Should this be a component of the proposed intervention? Yes/No

If yes, who should deliver this component of the intervention? *For example, GP, Pharmacist, Vascular Nurse*

What training would these staff (in the NHS AAA screening and surveillance programmes) need to deliver exercise advice?

What physical activity advice, if any, is currently given to those on your local AAA programme? *For example, just verbal, a leaflet, left to the GP entirely (if any)*

Are you able to refer individuals with AAA on to an exercise programme (e.g. phase 3 or phase 4 rehabilitation)?

If yes, what is the format of the exercise programme (e.g. group, home, digital, advice only)?

Please add any additional comments that you may have relating to exercise and physical activity.

**Advice on heart-healthy eating/weight management (where applicable)**

Should this be a component of the proposed intervention? Yes/No

If yes, who should deliver this component of the intervention? *For example, GP, Pharmacist, Vascular Nurse*

What training would these staff need to deliver dietary advice?

What dietary advice, if any, is currently given to those on your local AAA programme? *For example, just verbal, a leaflet*

Are you able to refer AAA patients on to a weight management programme (e.g. tier 2, tier 3 WM service)?

If yes, what is the format of the weight management programme (e.g. group, home, digital, advice only)?

Please add any additional comments that you may have on heart-healthy eating/weight management.

**Support for managing stress/anxiety related to living with AAA**

Should this be a component of the proposed intervention? Yes/No

If yes, who should deliver this component of the intervention? *For example, GP, Pharmacist, Vascular Nurse*

What support for managing clinical levels of anxiety is currently available to patients on your local AAA screening programme?

Who could/should deliver support for managing stress or (sub-clinical) anxiety for your AAA patients?

Please add any additional comments that you may have relating to stress/anxiety in this context.

**Support for managing low mood/depression in relation to living with AAA**

Should this be a component of the proposed intervention? Yes/No

If yes, who should deliver this component of the intervention? *For example, GP, Pharmacist, Vascular Nurse*

What support for managing clinical depression is currently available to patients on your local AAA screening programme?

Please add any additional comments that you may have regarding this element of the intervention.

**End of life planning support (for those who would like it)**

Should this be a component of the intervention? Yes/No

If yes, should all patients or only a specific group of patients be offered end of life support?

If only a specific group of patients should be offered that end of life support, how should this group be selected/identified?

Who should deliver end of life planning advice?

What (if any) existing support/service is there that you could refer (selected) AAA patients to for end of life planning?

Please add any additional comments that you may have relating to end of life support.

**Support for care givers (family and/or friends who are centrally involved in helping the patient to manage their condition at home)**

Should this be a component of the intervention? Yes/No

If yes, what kind of support should be included?

If yes, what format should be used? *For example, information-only, face to face advice, telephone helpline.*

Within your local AAA care pathway, what support is currently offered for care givers?

What are the key messages for carers or relatives/what information do they need?

Please add any additional comment that you may have relating to care givers’ support.

**Other intervention components**

Are there any intervention components which have been missed above? Yes/No

If yes, please tell us what else we could do

Overall, what are the key messages for men living with AAA? i.e. what messages do you believe would be useful in helping them to manage their condition and their overall cardiovascular health?

**Aim 2: To develop our eligibility criteria for the proposed intervention.**

Currently the proposed eligibility criteria for the intervention are: *Men with an abdominal aortic aneurysm identified through the NHS screening programme, who are not currently listed for surgery.*

Please answer the below questions taking these criteria into account.

Specificity of criteria

Are there any ways you think that the current criteria should be changed considering what the intervention is trying to achieve? Yes/No

If yes, please suggest how you would change the criteria (e.g. who would not be suitable?)

**Aim 3: To identify the best and most feasible ways to support patients to make these changes building on resources already available in the NHS and the AAA surveillance programme.**

***Format of the intervention***

At the moment, based on prior feedback from patients, families, and some experts in cardiovascular care we believe that the intervention we are planning to develop should include all of the following:

1. *An initial consultation between the individual with an AAA and a member of the aneurysm screening and surveillance team to assess cardiovascular risk factors, make a plan to address risk factors and build motivation for any patient behaviours needed to address risk*
2. *A self-help booklet for the individuals with an aneurysm which explains some basics surrounding cardiovascular care, as well as interactive planning and progress-tracking tools*
3. *Telephone-based follow-up tailored in terms of duration and content for each individual*

Do you agree with the above format? Yes/No

Do you have any other ideas you would like us to consider (e.g. would you remove or add anything)?

**Intervention delivery and level of support**

A number of possible intervention delivery formats are listed below.

Please score them out of ten for ‘likelihood of working’ and ‘ease of implementation.’

A score ten represents the best possible score and one the worst.

| Format | Likelihood of working | Ease of implementation |
| --- | --- | --- |
| Digital /’do it yourself’ (self-care) advice and planning support. |  |  |
| Paper based /self-care advice and planning support |  |  |
| 4-6 face to face meetings |  |  |
| 4-6 group meetings |  |  |
| Facilitated digital (e.g. 1 face to face meeting and 2-3 phone calls) |  |  |
| Facilitated paper based |  |  |
| Patients choose their preferred option (from any of the above) |  |  |

What members of AAA care staff are best placed to deliver the proposed intervention?

What training would these staff need?

**Building on what already exists**

**Based on your experience with/knowledge of your local AAA screening/surveillance programme:**

*If you cannot answer these questions (e.g. you do not have direct experience of the surveillance/screening practices in your area) please leave them blank.*

How many minutes does an individual spend with the screening staff at their first appointment?

How many minutes does an individual spend with the screening staff during surveillance appointments?

How many minutes do staff spend giving a patients who has just been diagnosed with an aneurysm advice regarding their cardiovascular health?

Would you have capacity in your screening/surveillance region to add more time for each patient’s appointment in order to address their cardiovascular risk-factors?

If NO, what do you think is necessary to be able to do that (e.g. more staff)?

How many contacts is a typical individual with an aneurysm currently likely to have with the AAA surveillance service in a year?

How long (in total across all contacts with the AAA service) is typically spent discussing self-care issues (as opposed to ultrasound results) during these contacts?

What current education about AAA self-care is delivered? (please describe what it includes)

Finally, is there anything else you think we should consider in designing a CV risk reduction service for people on the AAA screening/surveillance register?

***Thank you for taking the time to complete this questionnaire.***

***Your responses are greatly valued.***
