# Supplementary material for: Improving cardiovascular health in patients with an abdominal aortic aneurysm: development of the cardiovascular risk reduction in patients with aneurysms (CRISP) behaviour change intervention
Source: Pilot Feasibility Stud. 2024 Jan 29;10:17. doi: 10.1186/s40814-024-01445-z (PMC10823620; doi:10.1186/s40814-024-01445-z)
Supplement: Supplementary file 2 — Additional file 2: Appendix 2. Topic Guide. [file 40814_2024_1445_MOESM2_ESM.docx]

**Topic guide/Session plan: PPI session 1**

**22^nd^ October 2020 10am (Zoom)**

**90 minutes**

**Welcome (slides 1 and 2) 20 minutes**

Thanos to welcome all. Introduce ourselves. Then point out how PPI via Zoom can work better if you:

- Mute if you are not talking.
- Use the chat box to share thoughts as well if you wish.
- Feel free to switch camera off
- Use the hand up button

Give a brief overview of the study as a whole and how the PPI sessions slot in. Followed by the appropriate ‘housekeeping’ information.

Highlight the role of the PPI group in the development of the intervention. Also point out that this will be a very interactive process, feel free to share your opinion whilst also respecting others which may differ to yours.

Introductions of experts 1^st^. Then patients.

***Activity 1****: Go around the room and give everyone the opportunity to introduce themselves and briefly their experience of AAA and why they are here. Limit to a couple of sentences of similar.*

Housekeeping slide.

**What is an AAA (slides 3) 5 minutes**

Briefly explain what an AAA is.

**Aims (Slide 4 and 5) 2 minutes**

Explain the overall aim of the CRISP study and the aim of the session ‘to start developing the intervention.’

Also explain what an intervention is.

**Risk factors (slides 6) 5 minutes**

Discuss what risk factors are and those associated with AAA as well as cardiovascular disease. Highlight that not all risk factors are modifiable. Use the two graphs to highlight how making the right choices can affect ‘likelihood of having heart problems.’

**Greatest AAA concern (slide 7) 10 minutes**

Divide into two **breakout groups** if six or more participants are in attendance or three if more than 20 in attendance, for 10 minutes.

“Let’s now develop an intervention”

Give a bit of context about how the intervention could look. For example; ‘the expectation is that this intervention could be delivered by the AAA screening service as part of your screening appointment, along with some kind of online or telephone-based support.’

So, we now need to turn this basic idea into something specific that can be delivered to a wide range of different people with AAA.

***Activity 2****:* Ask participants what the previous information about risk factors means to them as someone with an AAA.

Prompts: What are you main concern living with an AAA? Are these risk factors important to you? What would be your initial reaction to being asked in one of your AAA screening appointments to discuss these risk factors and the possibility of making some changes in your lifestyle to reduce these risks? Probe further on any negative reactions (why do you say that?).

If they say anything interesting, could ask “can you tell me more about that” or similar to seek more in-depth information.

If someone is going off topic or giving a long, unhelpful response /telling you their life story, need to cut in with a brief summary /reflection and then ask a specific question to bring them back to the topic. Can also “I am aware of the time and want to know what other people think about this question”. If people have more to say on this, we would encourage to email it in to us, or hold it for later, when there will be another opportunity to say some more about any topic.

**Tea break (slide 8) 10 minutes**

*Come back to one group – friendly chat*

**Modifiable determinants (slide 9) 5 minutes (ONE GROUP)**

Initially we do the chatting, we give them some context (Tom best equipped to do this)

Discuss ‘barriers’ and ‘bridges’ to making behaviour change happen, using examples.

Taking increasing physical activity as an example suggest that a ‘barrier’ could be a lack of suitable facilities near where you live and a ‘bridge’ could be great transport links to your local leisure centre.

**Increasing physical activity (slides 10 and 11) 20 minutes**

**Different breakout groups again.**

Divide into breakout groups but ensure they are different to the first focus group, for 20 minutes.

***Activity 4****:* Let’s start with these questions.

**Barriers: Can anyone say what might stop you doing any of these things?**

What are the barriers to doing a bit more physical activity? [ This can be any kind of physical activity from walking to swimming to gardening to taking the grandchildren to the park ]

What are the barriers to walking outdoors?

What are the barriers to doing some muscle-strengthening exercises at home or at a gym

Read out the points on the slide (not all can read) and give them a minute to think. Encourage /remind them to add notes to the chat, as we are talking. 10 mins on barriers, then 10 on enablers.

*NB: Facilitators should keep the chat box open to see what is coming up and highlight any interesting issues to discuss further

*Prompts* (to be used or not, as needed):

Is where you live a barrier? If so, in what way?

Are you confident that you could increase the amount of physical activity or exercise you do? If not, why not?

How would other people in your life react if you decided to do more exercise?

What about motivation - Do you ‘want to do’ more physical activity or exercise? If not, why not?

What other things are more exciting/interesting or important than physical activity? (especially as a follow-on from the “I don’t have time” barrier)

How safe do you feel undertaking physical activity?

Is there anything else that might stop you doing physical activity? (resources /cost, knowledge /information about options, concerns /feelings)

**Enablers: Now let’s consider enablers for increasing physical activity:**

What might help you to do more physical activity?

What might help you to do more walking?

What would it take for you to be able to some muscle-strengthening exercises at home or at a gym?

*Prompts* (to be used or not, as needed):

What would persuade you that doing more exercise or physical activity is a good idea? (e.g. what would be the most important benefits if you did get more active? More control over your CV risk? Fitness /ability to get out and about and do things /ability to live independently; Having more energy /feeling good about yourself /better mood? What would it mean for other people in your life?

How could other people support you to do any of these things?

What would it take for you to feel safe undertaking some physical activity?

Overall, what kind of support would you need to get you going with any of these activities?

Would it easier to do ten two-minute bouts of activity over the course of a day (say walking on the spot, or doing some stretches) or one 20-minute bout of activity (like going out for a walk)?

**Come back together as one group.**

**Next steps (slide 12) 5 minutes**

Summarise what has been achieved and mention when the next meeting is and its aim.

We should also stress the importance of contacting us if they wish to – email & telephone.

**Keep meeting open if anyone wants to stay on to discuss any of the points raised – up to 2 hours. Feel free to leave, but we are happy to hang around for a while and talk about anything really.**
