# Supplementary material for: Improving cardiovascular health in patients with an abdominal aortic aneurysm: development of the cardiovascular risk reduction in patients with aneurysms (CRISP) behaviour change intervention
Source: Pilot Feasibility Stud. 2024 Jan 29;10:17. doi: 10.1186/s40814-024-01445-z (PMC10823620; doi:10.1186/s40814-024-01445-z)
Supplement: Supplementary file 3 — Additional file 3: Appendix 3. Risk Factor letter. [file 40814_2024_1445_MOESM3_ESM.pdf]

Tel: 0001111
E-mail: screening@screening.co.uk

Dear Mr Green

Thank you for coming along to the nurse assessment today. Please find below a summary of the results. These show your current heart health profile and (if relevant) actions that can be taken to improve it.

Based on your blood test results and measurements, currently your chance of having a heart attack, stroke or other heart problems in the next 10 years is: 27.5%. If there were 10 patients the same as you, around 3 of them would have heart problems within the next 10 years.

Your chance of developing a heart problem depends on the eight key heart health factors below. These have been placed into three categories:

- Red means you should take action now to reduce your risk.
- Amber means that some action would be helpful.
- Green means there is no need to take action (just keep going as you are).

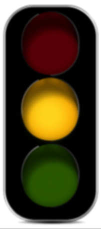

Smoking
To improve your heart health, you need to stop smoking.

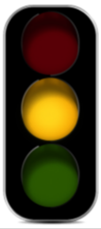

Physical Activity
To move to green, you need to undertake at least 180 minutes of “moderate intensity” physical activity each week, or spend less time sitting down.

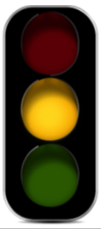

Weight
To move to green, you need to make some small changes to make your diet healthier. Doing more physical activity may also help.

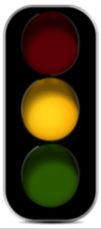

Diet
To move to green, you need to identify a few more small changes you can make to improve your diet and carry them out.

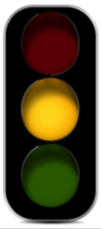

Alcohol
To move to green, you need to reduce your alcohol intake a little more. Have a look at our workbook on alcohol to find out how.

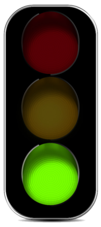

Stress
Great job - just keep going as you are.

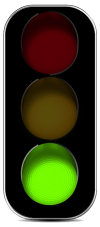

Low Mood
Great job – just keep going as you are.

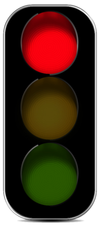

Blood Pressure
To move to amber, start taking steps to reduce your blood pressure. This can involve any of the above changes as well as taking medications.

Further information is available at screening.co.uk

Kind regards,

Your local aneurysm screening service
